# Iohexol plasma clearance simplified by Dried Blood Spot (DBS) sampling to measure renal function in conscious mice

**DOI:** 10.1038/s41598-021-83934-2

**Published:** 2021-02-25

**Authors:** Ana Elena Rodríguez-Rodríguez, Sergio Luis-Lima, Javier Donate-Correa, Laura Diaz-Martín, María Rosa Arnau, Alejandro Jiménez-Sosa, Flavio Gaspari, Alberto Ortiz, Esteban Porrini

**Affiliations:** 1 Research Unit, Hospital Universitario de Canarias, La Laguna, Tenerife, Spain; 2 Fundación General de la Universidad, Universidad de la Laguna, Tenerife, Spain; 3 Department of Nephrology and Hypertension, IIS-Fundación Jimenez Díaz, UAM, Madrid, Spain; 4 Fundación Canaria Investigación Sanitaria de Canarias (FIISC), La Laguna, Tenerife, Spain; 5 Servicio del Estabulario, Universidad de la Laguna, Tenerife, Spain; 6 Laboratorio de Función Renal, Instituto Tecnologías Biomédicas (ITB), Universidad de la Laguna, Tenerife, Spain; 7 Nephrology Department, Unidad de Ensayos Clinicos-UCICEC. Hospital Universitario de Canarias, Ofra s/n La Cuesta, 38320 La Laguna, S/C Tenerife, Spain; 8 Instituto Tecnologías Biomédicas (ITB), Universidad de la Laguna, Tenerife, Spain

**Keywords:** Nephrology, Kidney diseases

## Abstract

There is no simple method to measure glomerular filtration rate (GFR) in mice, which limits the use of mice in models of renal diseases. We aimed at simplifying the plasma clearance of iohexol in mice, using dried blood spot (DBS) sampling in order to reduce the amount of blood taken for analysis**.** GFR was measured simultaneously by *a reference method* in total blood—as described before—and *tested method* using DBS in fifteen male and six female C57BL/6J mice. Total blood extraction was 50 μL for the *reference methods* and 25μL for the *tested methods,* distributed in 5 samples. The agreement of GFR values between both methods was analyzed with the concordance correlation coefficient (CCC), total deviation index (TDI) and coverage probability (CP). The agreement between both methods was excellent, showing a TDI = 8.1%, which indicates that 90% of the GFR values obtained with DBS showed an error ranging from − 8 to + 8% of the reference method; a CCC of 0.996 (CI: 0.992), reflecting high precision and accuracy and a CP of 94 (CI: 83), indicating that 6% of the GFR values obtained with DBS had an error greater than 10% of the method in blood. So, both methods are interchangeable. DBS represent a major simplification of GFR measurement in mice. Also, DBS improves animal welfare by reducing the total blood required and refining the procedure.

## Introduction

Mouse models are very useful to study the pathogenesis of renal diseases. In these models, disease progression is generally evaluated by changes in renal histology and glomerular filtration rate (GFR). In mice, serum creatinine and 24 h creatinine clearance are unreliable in reflecting real renal function^1–5^. Thus, GFR must be evaluated by gold standard methods like the clearance of exogenous markers such as inulin (^3^H or ^14^C), ^51^Cr-EDTA, ^125^I iothalamate or iohexol^6,7^. Recently, our group developed a simple, reproducible and reliable method to measure GFR in conscious rodents by means of the plasma clearance iohexol^1,8^. In brief, this method consists in a single injection of 100 µL iohexol solution into a tail vein, followed by 5 blood extractions (~ 10 uL each), which then are diluted in water. Iohexol is then measured by HPLC–UV and the iohexol plasma clearance determined as the ratio between the dose of iohexol and the area under the curve^8^. This method offers several advantages such as the use of a non-radioactive contrast; mice are conscious and unrestricted which avoids unpredictable changes in GFR due to anesthesia; need of few samples and a total blood volume of 50 µL per procedure without vein catheterization. All this allows repeated measurements during the experiment and the assessment of GFR changes over time.

In humans, our group has recently simplified the plasma iohexol clearance using dried blood spot (DBS) sampling, a technique that requires a reduced volume of blood and simplifies pre-analytical processing^9^. We wanted to apply this simplification to the measurement of renal function in mice using the clearance of iohexol. Thus, we proposed to test the reliability of DBS sampling in mice without losing accuracy and precision compared with the standard procedure in total blood. Our hypothesis is that DBS sampling is a more simple approach to the measurement of GFR in mice by iohexol clearance and will help in reducing even more the total blood required per test, a relevant aspect of animal care in research.

## Results

GFR values using total blood samples averaged 351 ± 75 µL/min and 184 ± 101 µL/min for male and female groups, respectively. DBS testing showed mean GFR values of 353 ± 72 µL/min and 189 ± 100 µL/min for male and female animals, respectively (Table 1).

**Table 1:** GFR results with DBS compared with the reference method (total blood analysis).

Mice	Gender	GFR in total blood (µL/min)	GFR in DBS (µL/min)
1	Male	279	309
2	Male	310	317
3	Male	341	349
4	Male	255	254
5	Male	327	328
6	Male	352	354
7	Male	308	294
8	Male	334	323
9	Male	382	350
10	Male	334	344
11	Male	304	326
12	Male	430	408
13	Male	568	568
14	Male	342	348
15	Male	402	417
16	Female	71	77
17	Female	211	214
18	Female	193	207
19	Female	60	61
20	Female	308	305
21	Female	263	269

### Agreement between blood and DBS testing for GFR values

**Figure 1:**
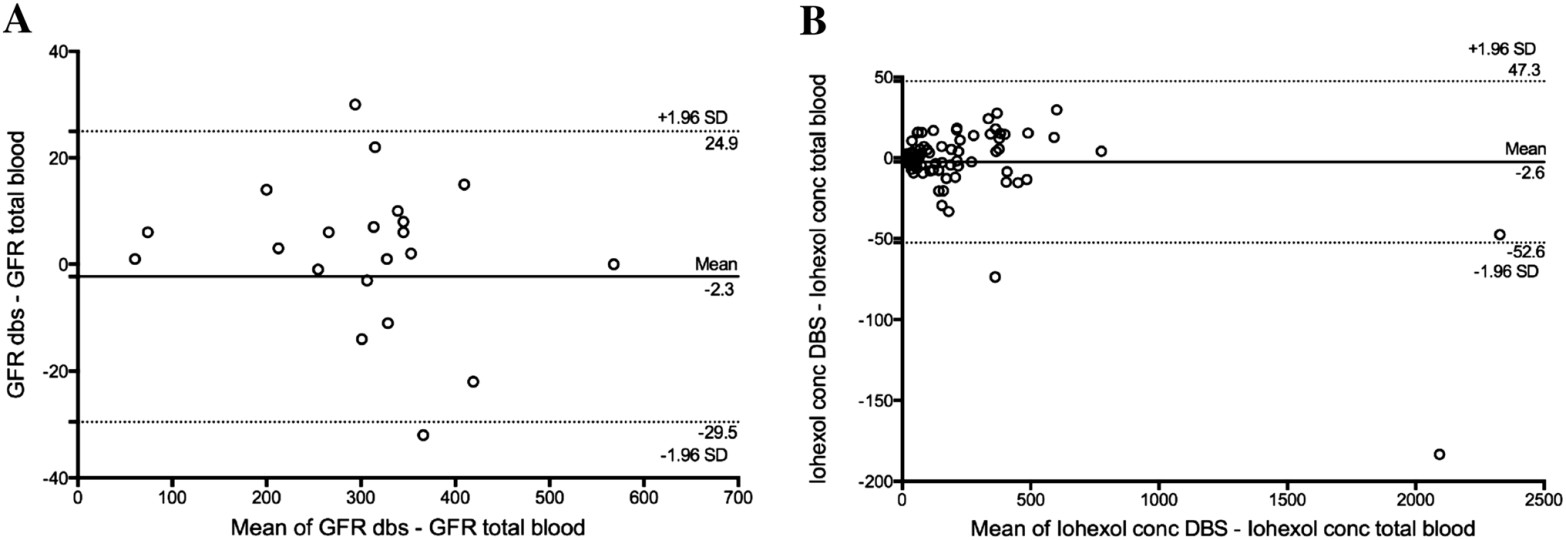
Bland–Altman plots and limits of agreement between (**A**) GFR values (µL/min) and (**B**) iohexol concentration values (µg/mL) both measured by the reference method in total blood and the tested method with DBS sampling. We used the GraphPad Prism software, version 6.0 (San Diego, CA; https://www.graphpad.com). Outliers did not change the good agreement between both methods.

The TDI was 8.1% (upper CI: 10.7), which means that 90% of the GFR values obtained with the tested method in DBS showed an error ranging from − 8 to + 8% when compared with the reference method in total blood. The CCC was of 0.996 (upper CI: 0.992) indicating high precision and accuracy of the tested method (DBS) with the reference method (total blood). Finally, the coverage probability (CP) was 94 (upper CI: 83), which indicates that only 6% of the GFR values obtained with DBS had an error range greater than 10% of the method in blood (Table 2). The Bland–Altman plot showed narrow limits of agreement between GFR values determined by the reference method in blood and the tested method in DBS: from − 29.5 to 24.9 µL/min, indicating very good agreement (Fig. 1A).

**Table 2:** Agreement analysis between the values of glomerular filtration rate values and iohexol concentrations measured by the tested method—DBS—and the reference method—total blood.

Statistics of agreement	GFR values (n = 21)	Iohexol concentration (n = 83)*
TDI (%)	8.13 (10.74)	17.43 (20.06)
CCC (%)	0.995 (0.992)	0.996 (0.995)
CP (%)	0.94 (0.83)	0.66 (0.60)

GFR: glomerular filtration rate, TDI: total deviation index; CCC: concordance correlation coefficient; CP: coverage provability. Upper CI: confidence interval is given for all values. *Points with large deviation from the curve are not included.

### Agreement between blood and DBS testing for iohexol concentrations

TDI was 17.4% (20.1), which means that 90% of the iohexol concentrations obtained with the tested method in DBS showed an error ranging from − 17 to + 17% when compared with the reference method in total blood. CCC was of 0.996 (0.995; upper confidence interval), reflecting high precision and accuracy of the DBS with the reference method. CP was 66 (60), which indicates that more than 33% of the iohexol concentrations obtained with DBS had an error range greater than 10% of the method in total blood (Table 2). The Bland–Altman plot showed narrow limits of agreement between the values of iohexol measured with the tested and the reference methods: from − 52.6 to 47.3 µg/mL, indicating very good agreement (Fig. 1B).

## Discussion

In this study we simplified the measurement of GFR in mice using the plasma clearance of iohexol replacing total blood samples for capillary blood deposited on filter paper—DBS sampling. The major finding of the study was that the clearances of iohexol using total blood or DBS were interchangeable as reflected by agreement analysis. This may be the consequence of the fact that iohexol is very stable at room temperature^10–12^ which led to comparable determinations of the molecule both in fresh and dried blood. Thus, DBS sampling represents a major simplification of the measurement of renal function by means of the clearance of iohexol in mice without losing accuracy and precision.

Creatinine is a limited marker of GFR in mice models^1–5^. In example, tubular secretion may account up to 30–50% of urinary creatinine, favoring GFR overestimation^2,3,5^. Thus, a reliable method to evaluate GFR in mice models is clearly needed. Several methods—both in mice and humans—have been developed to measure GFR after the injection of an exogenous marker. These involve the use of inulin (^3^H or ^14^C), ^51^Cr-EDTA, ^125^I iothalamate or iohexol^13–16^. The inulin clearance is neither simple nor practical: the standard technique, using radioactive (^3^H or ^14^C) labeled inulin, requires steady state blood marker concentration by using a continuous infusion and bladder catheterization, making necessary the use of anesthesia, which can affect GFR in an unpredictable manner. Thus, a single-bolus technique has been developed, followed by serial measurements over time^17^. Otherwise, non-radioactive methods based on fluorescently labeled inulin (FITC-Inulin) have been developed^18–20^. Nevertheless, the pre-analytical process for both labeled (^3^H, ^14^C) or FITC-inulin is extremely cumbersome, requiring several steps such as, dissolving the molecule in saline, which must be filtered, heated a high temperatures, dialyzed overnight to remove residual free radiolabel and lower molecular-weight fragments from inulin, and finally, the dialyzed inulin need to be filtered through a low-diameter pore membrane^17,20^. More recently, fluorescein-labeled sinistrin, a soluble polyfructosan has been proposed as an alternative method to measure renal function^21^. Finally, a new method to measure GFR by means of a transcutaneous device has been developed. This method relies on a device that permits the transcutaneous measurement of the elimination of the fluorescent marker FITC-sinistrin^22–25^. However, some limitations rise concern about the reliability of this approach. Firstly, the marker is not directly measured and the kinetic analysis is estimated through the change in relative fluorescence intensity over time. Thus, conversion factors are needed to estimate the GFR value in mL/min, which may lead to uncertainty of GFR results^25^. In fact, the agreement of GFR measured with transcutaneous device versus the reference method in plasma was poor for both the two and one compartment analysis: *r*^*2*^ 0.33 or 0.42, respectively^25^. Another recent study^26^ validated the transcutaneous with the plasma clearance method in lean and obese C57BL/6 J mice showing a weak correlation (*R*^*2*^ 0.704) in lean animals and very low performance in obese mice, assessed by very wide limits of agreement^26^. Finally, the cost of the transcutaneous method which about US$1750 per device^27^ is a point to consider, which may limits its use in experiments with a large number of animals.

In comparison, the method using the plasma clearance of iohexol with DBS sampling, which we proposed, has been tested with the reference method in plasma, showing excellent agreement with acceptable accuracy and precision. Also, the pre-analytical and analytical phases are simple and reproducible. Of note, the procedure is cheaper compared to others^28^. Another point relevant to consider is the agreement between the plasma clearance of iohexol and other methods used to measure GFR. As indicated above, the clearance of inulin has been considered as the gold standard to evaluate GFR. However, this is more an historical fact, since inulin the first method described. To the best of our knowledge, there is no evidence of the method to which insulin was confronted with to ascertain its status as *the* gold standard. Also, some reports indicated a relevant extra renal clearance of inulin, which was higher than the other methods (iohexol, DPTA, EDTA)^29^. This suggests that the clearance of inulin may actually overestimate real GFR. In any case, the agreement between the plasma clearance of iohexol and that of inulin proved to be excellent both in humans and rodents. Sterner et al., in humans showed that using 5 point determinations in analysis lead to comparable results in inulin and iohexol plasma clearances^30^. In the same line, Turner et al. evaluated in the same group of rats the plasma clearances of iohexol and inulin showing that both methods had excellent agreement as indicated by a 15% accuracy of 82.3% and narrow limits of agreement^20^. Similar results have been observed in rats in a model of AKI^31^. So, we may conclude that both methods are comparable in reflecting GFR.

In small animals reducing to a minimum blood extraction is crucial. The average total blood volume of a mouse is about 78 mL/kg, i.e. 1.56 mL for a 20 g mouse^32,33^ and approximately 0.16 mL of blood from a 20 g mouse can be safely withdrawn. In case of needing multiple samples taken at short intervals, smaller volumes should be removed each time to maintain the physiological stability of a mouse. The amount of blood withdrawn per week should not exceed 7.5% of the total blood volume^34^. The DBS method reduces the total amount of blood required by half compared to reference method i.e. from 50 to 25 µL per procedure, improving animal-welfare and integrity in accordance with Russell and Burch’s 3Rs model for animal research^35^. Just as important, we refined the original procedure and we helped reducing the number of animal needed for research, since GFR can be measured in the same animal repeatedly. Other advantages of the DBS method include that there is no need of tubes for blood collection or cold-storage facilities since DBS is stable at room temperature. All these factors reduce the cost of the procedure and the experiment. Finally, there is no specific mailing restriction for dried blood samples, which facilitates the shipment of samples between laboratories.

The main limitation of this method is possible extravasations at iohexol injection. Having staff with high experience in animal management with good injection technique can solve this. However, the procedure can be repeated one day apart since the half-life of iohexol elimination is very short (approximately 120 min)^36^. Among the strengths, the method was validated for both male and female mice, which represents not only potential differences related to sex, but also a different range of GFR values. Validation for both sexes is in line with research funding agency requirements for research with animals.

Finally, our method represents a relevant methodological simplification to study renal function in small animals without losing precision and improving animal welfare.

## Material and methods

### Animals

A total of 15 male and 6 female C57BL/6J healthy mice were used for this study^37^. Animals of approximately 8 weeks of age were housed in a same room at temperature of 22 ± 2 °C, relative humidity of 50 ± 15% with food and water ad libitum in the animal facilities of the University of La Laguna. Animal care was performed in accordance with ARRIVE (*Animal Research: Reporting of *In Vivo* Experiments*) guidelines^38,39^, and institutional guidelines in compliance with Spanish (Real Decreto 53/2013, February 1. BOE, February 8, 2013, n: 34, pp. 11370–11421) and international laws and policies (Directive 2010/63/EU of the European Parliament and of the Council of 22 September 2010 on the protection of animals used for scientific purposes) and were approved by the Institutional Animal Care and Use Committee (Comité de Ética de la Investigación y de Bienestar Animal (CEIBA) of University of La Laguna, Spain).

### Experimental design: clearance of iohexol in total blood-reference method and DBS samples-tested method

**Figure 2:**
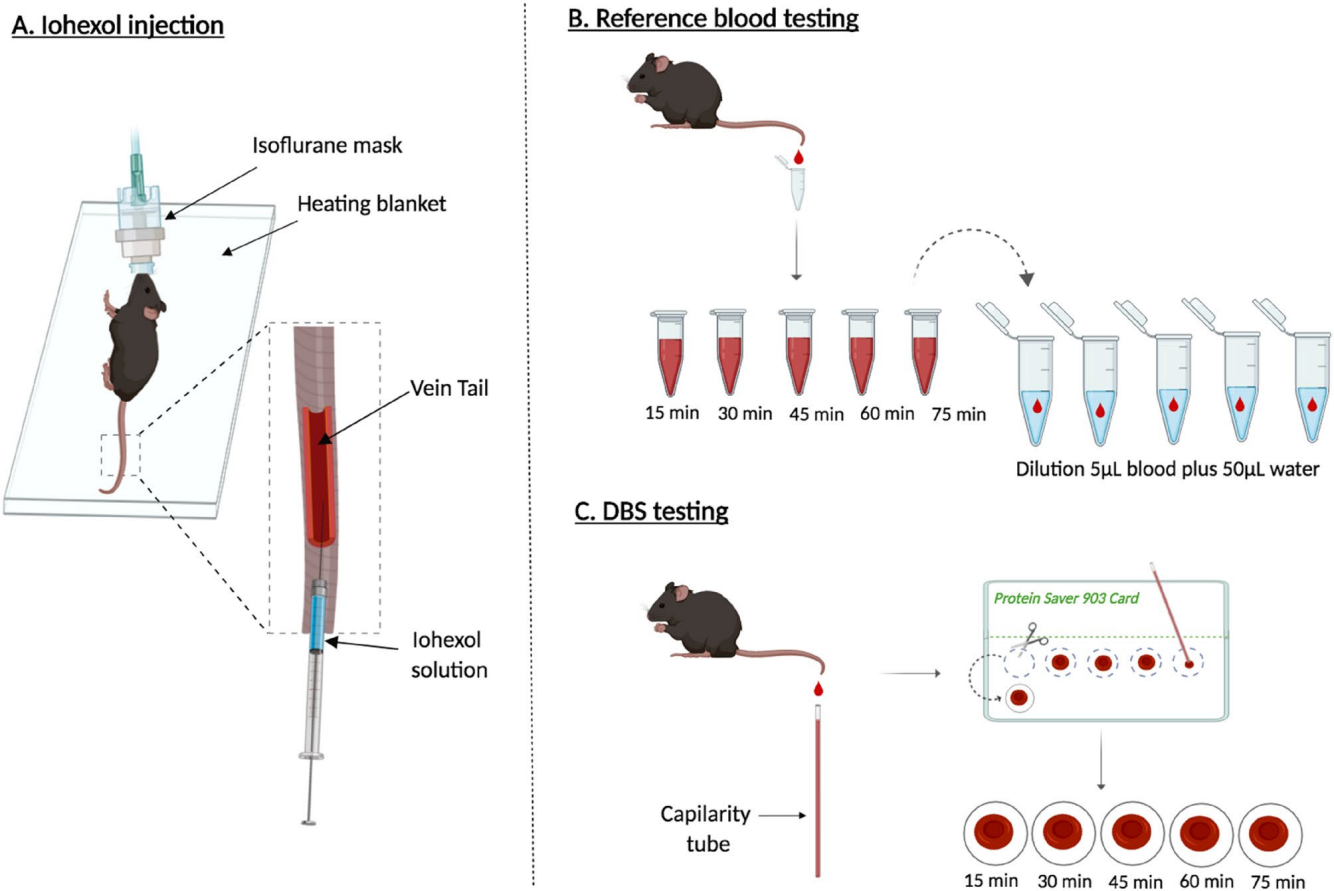
Experimental design. Male and female mice were used. (**A**) *Iohexol injection*: after slight sedation with isoflurane, 100 µL of iohexol solution mice were injected intravenously into the tail vein. (**B**) *Reference blood testing:* 15, 30,45, 60 and 75 min after injection a drop of blood (~ 10 µL) were collected from the tip of the tail and deposited in a tube. Then. 5 µL are taken from the tube and diluted in 50 µL of water. (**C**) *DBS testing*: at the same times**,** exactly 5 µL of blood were taken using heparinized capillary tubes and deposited in filter paper. Then, after 24 h drying, samples are cut-off for analysis. *Figure designed and made by Dr. Ana Elena Rodriguez-Rodriguez using the Biorender application in its free version (*https://app.biorender.com*).*

We compared the GFR values determined simultaneously by the plasma clearance of iohexol using total blood and DBS samples in the same group of animals (Fig. 2). Animals were weighed before the GFR procedure. Then, mice were slightly sedated with isoflurane (2.5%) administered by facemask only during injection. The solution of iohexol injection was prepared from Omnipaque 300 (GE Healthcare) by diluting with saline 1:10 to make a concentrated solution at 64.7 mg/mL. A total of 100 µL of this iohexol solution was injected intravenously into the tail vein, corresponding to a dose of 6.47 mg of iohexol (Fig. 2A). The tail tip (~ 0.5 mm) was cut before injection to extract ~ 15 µL of blood to measure haematocrit.

*Reference method in total blood*: at 15, 30,45, 60 and 75 min after injection a drop (~ 10 µL) of blood is extracted from the tip of the tail and deposited in an empty tube (Fig. 2B).

*Tested method in DBS*: 5 µL of blood are taken at the same time points, using heparinized capillary tubes (Hirschmann, Supplier Article Number: 9000205; https://www.hirschmann-laborgeraete.de/en/artikelgruppe/90002?parent={9891EE8A-238C-4992-8921-7BC349E503B)) and deposited on filter paper (Whatman 903, GE Healthcare, Cardiff, UK) (Fig. 2C).

For blood extractions, no further sedation was necessary, and the animals were conscious and unrestricted during the whole procedure.

### Sample preparation

#### Reference method in total blood

5 μL of total blood are taken from the tubes of each time-point extraction by a standard pipette and added to a tube containing 50 μL distilled water (Fig. 2B). Then, these samples are deproteinized with 50 μL perchloric acid 5%, vortexed and centrifuged for 10 min at 10 min at 20,000*g*. Finally, an aliquot of 15 μL from the supernatant is injected in the HPLC system for analysis.

#### Tested method in DBS

5 μL of blood—taken with a capillary tube—were placed onto filter paper and allowed to dry for at least 24 h. The extraction of iohexol from DBS samples is based on a previous publication of our group^9^. In brief, the DBS sample is punched out from the paper (containing the whole amount of blood), placed in a tube with 200 µL of 5% perchloric acid, deproteinized by 3 min of vortex mixing, ultrasonicated for 15 min and incubated at room temperature for 30 min. Finally, the tubes are centrifuged at 20,000*g* for 10 min and an aliquot of 60 μL from the supernatant is injected in the HPLC system for analysis.

### Iohexol measurement

A volume of 15 µL of the supernatant for *total blood analysis* or 60 μL for *DBS testing* for each sample was chromatographed by a C18 reverse phase column (5 µm, 150 × 4.6 mm, Advanced Chromatography Technologies LTD, Scotland) using a HPLC system (Agilent series 1260 Infinity—Agilent Technologies, Santa Clara, CA, USA) equipped with a diode array detector set at 254 nm^40^. Iohexol isomers were eluted by a mixture of deionized water/acetonitrile (96:4 by volume, adjusted to pH 2.5 with phosphoric acid) pumped at 1.0 mL/min flow rate. Internal calibration curves of iohexol were prepared for each set of samples.

### Pharmacokinetic analyses

GFR was calculated by means of a one-compartment model considering only the elimination phase of the iohexol and using five sampling points. We used blood instead of plasma samples since iohexol is quantitatively distributed to the plasma compartment as demonstrated by Krutzén et al.^41^. Plasma concentrations of iohexol were recalculated from blood levels using the formula: Cplasma = Cblood/1 − Hct where Hct is the hematocrit. Hematocrits were determined using the formula: Hct = (H1/H2)*100 where H1 is the height of the red blood cell (RBCs) column, and H2 is the height of the RBC plus the height of the plasma column after centrifuging an heparinized capillary filled of blood at 10 min at 4500*g*. So, the concentrations of iohexol at 15, 30, 45, 60 and 75 min were fitted by a slope-intercept method to calculate the area under the curve (AUC). The slope intercept approach uses data only of the slow exponential and the fit is done by taking the natural logarithm of the plasma concentrations (Pi). The linear regression of ln(Pi) against time is performed to determine the slope, − k, and the intercept, ln(P0). The AUC of the single exponential is given by: AUC = (P0)/k. The iohexol plasma clearance was determined as the ratio between dose of iohexol and AUC (dose/AUC) after applying a correction factor of 0.89 based on a previous publication of our group where we found that the simplified method (one-compartment model) overestimated the GFR in about 11% with respect to the reference method (two-compartment model)^8^.

### Statistical analysis: test of agreement

The agreement between the concentration of iohexol and GFR values calculated using blood and DBS samples was evaluated by statistics of agreement for continuous variables including the limits of agreement described by Bland and Altman^42^, the total deviation index (TDI), concordance correlation coefficient (CCC), and coverage probability (CP) as proposed by Lin et al.^43^. The limits of agreement are a simple graphic tool that describes the limits that include the majority of the differences between two measurements. The narrower these limits are, the better the agreement. CCC combines elements of accuracy and precision. Its scores range from 0 to 1 and a value > 0.90 reflects optimal concordance between measurements. TDI is a measure that captures a large proportion of data within a boundary for allowed differences between two measurements^43^. CP ranges from 0 to 1; it is a statistic that estimates whether a given TDI is less than a pre-specified percentage^44^. The ideal situation is to have a TDI < 10%, meaning that 90% of the estimations fall within an error of ± 10% from the gold standard.

For the Bland and Altman test and figure, we used the GraphPad Prism software, version 6.0 (San Diego, CA; https://www.graphpad.com). For the agreement analyses, we used the statistical package AGP (Agreement Program version 1.0 (IGEKO, SP) available at: www.ecihucan.es/lfr/apps/?dir=agreement_installer. The AGP is based on the R code originally developed by Lawrence Lin^43^. The AGP was developed to simplify the use of the tool given in the R agreement package (R Core Team (2017). R: A language and environment for statistical computing. R Foundation for Statistical Computing, Vienna, Austria; http://www.r-project.org/index.html). Results were expressed as mean ± SD.
